# [Retracted] MicroRNA‑873 serves a critical role in human cervical cancer proliferation and metastasis via regulating glioma‑associated oncogene homolog 1

**DOI:** 10.3892/etm.2024.12451

**Published:** 2024-02-26

**Authors:** Juan Feng, Tingfeng Wang

Exp Ther Med 19:1243–1250, 2020; DOI: 10.3892/etm.2019.8348

Following the publication of the above article, it was drawn to the Editors’ attention by a concerned reader that certain of the colony formation assay data shown in Fig. 2C and E, and the cell migration and invasion data shown in Fig. 3A and B were strikingly similar to data appearing in different form in other articles written by different authors at different research institutes that had already been submitted for publication elsewhere prior to the submission of this paper to *Experimental and Therapeutic Medicine* (certain of those articles have now been retracted). Owing to the fact that the contentious data had already apparently been submitted for publication prior to the submission of the above article to *Experimental and Therapeutic Medicine,* the Editor has decided that it should be retracted from the Journal. The authors were asked for an explanation to account for these concerns, but the Editorial Office did not receive a reply. The Editor apologizes to the readership for any inconvenience caused.

